# Factors Associated with Linkage to Care following Community-Level Identification of HIV-Positive Clients in Lira District

**DOI:** 10.1155/2022/4731006

**Published:** 2022-02-04

**Authors:** Claire Adwar, Steven Sean Puleh, Isaac Ochaba, Isaac Ogweng, Deo Benyumiza, Kosta Amusu, Brenda Achola, Francis Ocen, Lydia Abolo, Edward Kumakech, Celestino Obua

**Affiliations:** 1 Lira University, Faculty of Health Sciences, Department of Public Health, Lira, Uganda; 2 Lira University, Faculty of Health Sciences, Department of Midwifery, Lira, Uganda; 3 Mbarara University of Science and Technology, Mbarara, Uganda

## Abstract

**Background.:**

Community HIV testing helps to increase access to high-risk groups who are less likely to visit a clinic for a test. A large proportion of people identified with HIV following community-based testing are not easily linked to care compared to facility-based identified cases. There is a paucity of literature on linkage to HIV care and its predictors particularly following community-based testing in a rural setting. We assessed the level of linkage to the care of HIV-positive individuals and associated factors following community-level identification in Lira district.

**Method.:**

A cross-sectional survey was conducted in Lira district employing mixed methods among HIV-positive adults identified in the communities. Quantitative data were collected from 329 randomly selected study participants using interviewer-administered questionnaires. Key informant interview guide was used to collect qualitative data. The data were double entered, cleaned, and analyzed using SPSS version 23. Odds ratios and confidence intervals were used to assess the association between predictors of linkage with HIV care. Qualitative data were analyzed using thematic content analysis.

**Results.:**

The respondents were aged between 18 and 85 years with a mean age of 42.9 (SD = 11.6). The level of linkage to HIV care following community-level identification of HIV testing in Lira district was 98% (95% CI 96.07–99.33). Clients who self-initiated the HIV testing were more likely to link to HIV care than their counterparts (AOR = 9.03; 95% CI 1.271–64.218, *p* = 0.028). Key informants identified factors influencing linkage to care as health education, counseling, follow-up, and family support. Fear of stigma, disclosure, denial, and distance to facility were reported as barriers to linkage.

**Conclusion/Recommendation.:**

The level of linkage to HIV care following community identification was found to be excellent (98%). Predictors to linkage to care included self-initiated testing, positive perception of distance, and waiting time at health facilities. We recommend health education, counseling, follow-up, and family support as interventions to strengthen successfully linking to care.

## 1. Introduction

Advancements in antiretroviral therapy (ART) and HIV care over the last 3 decades have contributed substantially to transforming the HIV epidemic into a less fatal public health threat than it posed in earlier years of the epidemic [1]. Several studies have established the potential benefits of expedited linkage to and retention in HIV care for persons diagnosed with HIV such as lower rates of viral resistance, earlier viral suppression and lower likelihood of transmission, lower mortality, and better overall quality of life [2–4]. These benefits are only possible within a robust continuum of care where newly diagnosed persons are promptly linked to HIV care.

In efforts to end the HIV/AIDS epidemic, stakeholders at the global level have set several targets towards achieving universal testing and treatment of people living with HIV. United Nations member states under the UNAIDS program established the “95-95-95” target, which aims to diagnose 95% of people living with HIV, provide treatment to 95% of people diagnosed, and achieve viral suppression in 95% of people on treatment by 2030 on a global scale [1]. As part of the global agenda to accelerate action towards this cause, current approaches to increase testing uptake have focused on broadening HIV Counseling and Testing (HCT) options through community-based methods [5]. In Uganda, community testing has been implemented through home-based testing and counseling, outreaches, workplace-based testing, “moonlight” testing, and HIV self-testing [6]. Several studies have reported successful implementation and high uptake of community-based modalities for HCTin sub-Saharan Africa compared to health facility-based HCT [7–9]. This has provided opportunities for early diagnosis of HIV-positive individuals who may otherwise present to health facilities late in the course of illness. However, the success of community-based HCT (CB-HCT) is contingent on the efficient linkage of diagnosed persons to HIV care. A critical setback of community-based testing is that individuals who are tested outside health facilities generally have lower rates of linking to care and initiation of ART than those who are tested at health facilities [8, 10].

Given the current “test and treat” recommendations for adults who test positive for HIV irrespective of CD4 count or clinical stage [11], understanding the dynamics of linkage is particularly important. Although national and international guidelines consider prompt linkage to care to be within a month, in this study, linkage to care was defined as having been initiated on ARTfollowing diagnosis within a 6-month period because of COVID-19-related restrictions. Extended periods for linkage to care have been used in other studies in settings elsewhere [12, 13]. Notably, extended periods of linkage to HIV care may be required in community HIV testing programs where community health workers are conducting the testing, and antiretroviral (ARV) treatment may not be immediately available. A recent meta-analysis identified few studies conducted in sub-Saharan Africa and reported substantial variability in levels of linkage following community HCT ranging from as low as 10% to 79% within 12 months of diagnosis [14]. In Uganda, linkage to care in the general population has not been well characterized as studies have either been done under program conditions or focused on key populations such as fisherfolk communities, female sex workers, and refugee settlements, and low levels of linkage have been described. In these settings, the most prominent barriers to seeking care cited among participants were fear of stigma, cost of transport, and prohibitive schedules [12, 15, 16]. However, there is a paucity of research on linkage to HIV care and associated factors particularly following community-based testing in rural settings.

## 2. Methods

### 2.1. Study Design/Setting.

This study employed a descriptive cross-sectional study design using quantitative and qualitative approaches (mixed methods) to collect data in August 2020. This study was conducted in communities that are within the catchment area of the three health facilities; Ogur HC-IV, Aromo HC-III, and Barapwo HC-III. These facilities are located in Erute, North County, Lira District, about 340 kilometers north of Kampala, Uganda’s capital. In this setting, community-based HCT is conducted by the health workers from the nearest health facilities who make community visits to conduct HIV testing in schools, markets, places of worship, and other places of social gathering. These are places where key populations are likely to be reached by testing [17]. The tested client’s information is registered in HCT registers, transferred, and kept at the health facilities. Fortunately, every community has community health personnel called HIV linkage facilitators who ensure that those who test positive are counseled and linked to HIV care.

### 2.2. Study Population.

The study included HIV-positive adults (18 years old) who had been diagnosed through community-based HIV testing services within the previous 6 months. To explore the perceptions about linkage and its influencing factors using qualitative data, we conducted the study among community linkage facilitators, ART clinic in-charges (nurses or clinical officers), and counselors at the selected health facilities in Lira district.

### 2.3. Sample Size Estimation.

The sample size for our study was estimated following the methods for estimating sample size for cross-sectional studies by Kish and Leslie [18] to determine the level of linkage and its associated factors. The elements that were entered into the formula to calculate the sample size were *Z* = 1.96 at 95% confidence level, *e* = allowable error of 5%, and *p* = proportion of HIV-positive clients from the community who were successfully linked to care at 69% [19] and an estimated sample size of 329 participants was obtained. To explore perceptions of the healthcare workers in the HIV care continuum about linkage to care and influencing factors using qualitative design, a sample size of 10 key healthcare workers was determined by data saturation point [20].

### 2.4. Sampling Criteria.

In this study, the list and contact information of people who tested HIV positive in the community was obtained from the HCT registers at the health facilities. We used stratified random sampling techniques whereby a proportionate number of participants from the list that was disaggregated by the three health facilities were selected. The eligible participants were then contacted by phone to make an appointment for a home visit to collect the data. The linkage facilitators at each of the health facilities assisted in locating the selected participants in the communities.

We conducted purposive sampling among key health workers in the HIV care continuum who included ART clinic managers, counselors, and linkage facilitators to obtain qualitative data. Interviews were conducted until saturation was reached. The sampling and data collection from the selected participants was completed over a period of one month (August 2020).

### 2.5. Data Collection Tools.

We used a questionnaire and an interview guide to collect data. In order to ensure comprehensiveness and depth of research findings, an interview method is sufficient to triangulate, complement, and deepen the findings from the questionnaire on some key items. The questionnaire was used to collect data on whether the HIV-positive patients were (a) initiated on ART following their community-level diagnosis, (b) provided pretest counseling, posttest counseling, and health education, (c) experiencing challenges in accessing healthcare such as transportation costs, distance to the facility, and unfair treatment by health workers, and (d) experiencing physical signs of ill health. The interview guide asked the healthcare workers about their (a) perceptions of the influencers and barriers to linkage; (b) how they ensured that those who tested positive in the community were linked to care; and (c) what challenges they faced in linking clients to care. The tools were developed from the local literature and pretested among 30 participants in Ober HC-III, a nonparticipating facility in the study, to evaluate the properties of the tools prior to use in data collection. The properties of the questionnaire, especially the reliability index Cronbach alpha, were good at 0.8.

### 2.6. Study Variables.

In light of the evidence that community-based methods identify a significantly higher number of HIV-positive cases than facility-based HCT [8], there is a need to fill a critical gap in knowledge of the underlying influences of linkage following CB-HCT to leverage its full benefits. This study sought to determine the level of linkage to HIV care following community-based HCT and investigated patient-related factors (age, sex, marital status, employment status, education level, fear of unfair health workers, and condom use) as well as healthcare provider perspectives on linkage to care within the general population in rural Northern Uganda. Other variables investigated were health systems factors (distance to the health facility, provision of pre- and posttest counseling, provision of health education, local economy, and cost of transport). These variables were selected for the study based on the health behavior change model for HIV prevention and care. The model assesses personal factors, community-level factors, and health systems factors that influence HIV risk or care [21].

### 2.7. Data Collection.

Quantitative data were collected using interviewer-administered questionnaires, and an interview guide was used to gather information from key healthcare providers in the HIV care cascade. Written consent was sought from all participants before screening to determine their eligibility to participate in the survey. The interviewer-administered questionnaires were administered to the participants in the local language (Lou) in turns until the required samples were reached per health facility. These interviews took about 10–20 minutes. The precoded responses were translated into English on the spot during data entry. Consenting healthcare providers were interviewed using a key informant interview guide to collect qualitative data, and interviews lasted between 20 and 25 minutes. Key informant interviews were conducted in English language as all healthcare providers are fluent in English language. The interviews were audio-recorded. The audio recordings in English language were transcribed verbatim before analysis.

The questionnaires and key informant interviews were conducted by three (03) interviewers with a health background (Public Health, Midwifery and Community Psychology and Psychotherapy). They were trained by Mbarara University of Science and Technology on concept and proposal development, data collection, analysis, and responsible conduct of research over a period of 15 days.

### 2.8. Data Management and Analysis.

We reviewed the study tools at the end of each data collection day to ensure the completeness of the data collected. The data entry screen was created in SPSS version 23 with consistency checks. The study team double entered the data before transferring it to STATA version 14 for analysis. Whereas EpiData is the preferred software for data entry and management, we used SPSS to allow for testing of the psychometric properties of the locally developed questionnaire, and we avoided data entry errors through double data entry, double-entry validation analysis, and correction of discrepancies before running statistical analysis. The linkage to care being a categorical variable was analyzed at a univariate level with frequency counts, percentages, and the 95% confidence interval, and a 5% significance level was calculated. Bivariate and multivariate analyses were conducted to identify predictors of successful linkage to HIV care. At the bivariate level, associations were sought between linkage to care and its potential predictors (education level, marital status, health education, post- and pretest counseling, whether the client has a committed sexual partner, self-initiation of testing, and experience of signs and symptoms of ill health) using logistic regression expressed as a crude odds ratio at a 95% confidence level with a 5% significance level. Variables with associations at *p* = 0.2 in bivariate analysis (education level, self-initiated testing, distance to the facility, and fear of unfair treatment by health workers) were significant at p 0.2 in multivariate analysis and were expressed as an adjusted odds ratio at a 95% confidence interval. Variables with associations at *p* > 0.2 were excluded from the multivariate regression model (experiences of signs and symptoms of ill health, receipt of pre/posttest counseling, and marital status). Qualitative data were analyzed manually using thematic content analysis according to Braun and Clarke [22], which describes seven steps of data analysis, including (1) transcription, (2) reading and familiarization, (3) coding, (4) searching for themes, (5) review of themes, (6) naming the themes, and (7) finalizing the analysis and interpretation of the results.

### 2.9. Ethical Consideration.

The study protocol was reviewed, and clearance was provided by the institutional review board of Gulu University (GUREC-049-20). The protocol was further cleared for collection of data in Uganda by the national research regulator, Uganda National Council of Science and Technology (RESCLEAR/01 as of July 13, 2020).

### 2.10. Administrative Clearance.

Administrative clearance was obtained from the chief administrative officer of Lira, the District Health Officer of Lira district, and the managers of the respective health facilities of Ogur HC-IV, Barapwo HC-III, and Aromo HC-III.

## 3. Results

In this study, the respondents were aged 18–85, with a mean age of 42.9 years (SD ± 11.6). The majority of the respondents (70.8%, 233/329) were females. The majority of the respondents (62%, 204/329) had completed primary level education. In addition, most of the respondents (85.7%, 282/329) were peasant farmers (Table 1).

### 3.1. Association of Sociodemographic Characteristics and Linkage to Care following Community-Level Identification.

In our study, the respondents who were females were 1.98 times more likely to be linked to care compared to the males; however, the association was not statistically significant (COR = 1.98; 95% CI 0.52–7.55, *p* = 0.32). In addition, the respondents who were married were 1.8 times more likely to be linked to care compared to the singles, but the association was not statistically significant (COR = 1.80; 95% CI 0.48–6.84, *p* = 0.39) (Table 2).

### 3.2. Linkage to HIV Care.

From the quantitative data, the linkage was assessed among 329 participants in three public health facilities. The level of linkage to HIV care following community-level identification was 98% (95% CI 96.07–99.33).

From the qualitative analysis, the high level of linkage could be attributed to community ART initiation and health education by the healthcare providers in the community. Availability of drugs and ART registers during outreaches ensured immediate and early initiation to care. This innovation helped improve the level of linkage following community diagnosis. In addition, the registers help in capturing client information that would ease follow-up after ART initiation. Community ART initiation also helped to reduce the burden of transport cost and lessen the loss of clients after diagnosis. One of the counselors said that “We usually carry drugs and our register to the community so that the client who accepts to be initiated on ART is initiated there and then.” A 27-year-old female, counselor.

In addition, the high linkage level can also be attributed to the health education programs in the communities. Most of the key informants (60%) suggested that creating awareness through giving health talks provides clients with information on the importance of knowing their HIV status and early initiation on ART. This helped in guiding their decisions and increasing rates at which clients linked to care. Health talks given were mainly on HIV prevention, care, and treatment. One respondent said, “Because you will have explained and digested everything with the client, they pick all the information relevant for them to know what is right and what is not right.” Counselor at Aromo HC-III.

Our study showed that the majority of the respondents (67.2%, 215/329) reported the cost of transport as a major barrier to linkage to HIV care. Additionally, clients who received posttest counseling were 34 times more likely to be linked to care compared to their counterparts who did not (COR = 34, 95% CI 17.696 to 66.611, *p* ≤ 0.001). Clients who had a steady sexual partner were 0.5 times less likely to be linked to HIV care (COR = 0.5, 95% CI 0.137 to 1.972, *p* = 0.34). However, the association was not statistically significant. On the other hand, clients who perceived distance to the health facility as a barrier were 0.4 times less likely to be linked to HIV care (COR = 0.4, 95% CI 0.118–1.704); however, the association was not statistically significant (Table 3).

Logistic regression was conducted to find out the association between independent variables in both Tables 3 and 4.

### 3.3. Multivariable Logistic Regression Model for Linkage to HIV Care following Community Diagnosis.

In this study, clients who initiated the HIV testing were 9 times more likely to be linked to HIV care compared to those who did not (AOR = 9.03; 95% CI 1.271 64.218, *p* = 0.028). On the other hand, clients who had attained at least secondary level education were 0.03 times less likely to be linked to HIV care compared to those who did not have any education at all (AOR = 0.03; 95% CI 0.002 0.451, *p* = 0.011).

### 3.4. Qualitative Results.

Five themes emerged as facilitators to linkage to care: health education, counseling, follow-up, family support, and on-site initiation.

### 3.5. Health Education of Clients.

Of the 10 key informants, 60% (6/10) reported that creating awareness through giving health talks provided clients with information on the importance of knowing their status and early initiation of ART. This innovation plays a major role in guiding their decisions and thus increasing the rate at which clients are linked to care. The health talks given were mainly on HIV prevention, care, and treatment. One respondent said, “Because you will have explained and digested everything with the client, they pick all the information relevant for them to know what is right and what is not right.” A 35-years-old male, counselor.

### 3.6. Counseling.

The diagnosis of HIV can generate a lot of anxiety and mental pressure at an individual level. Counseling helps prepare patients for their seropositive and seronegative status. In addition, counseling helps in fixing anxiety, denial, anger, and guilt associated with testing positive. Because patients come from all walks of life and HIV infection is lifelong, counseling was done before and after testing. Counseling of the participants did not only prepare the clients psychologically but also increase the rate at which clients accepted their results. In this study, many participants suggested that counseling be done thoroughly by trained counselors. During counseling, clients were told the dangers of not being on ART, which included the virus replicating in one’s body in large amounts, increasing the risks of being susceptible to many diseases. Intense counseling, therefore, was vital in ensuring that clients were linked to care. One Linkage facilitator reported that

“Before clients are counselled, they have a certain mood, but after counseling, they feel alright and accept what we tell them.” A 38-year-old female, linkage facilitator.

### 3.7. Follow-Up.

Following up with clients is a very important way of monitoring retention in care in order to ensure the long-term success of ART therapy. A majority of the key informants suggested follow-up as a way of ensuring that those who test positive are linked to care. Those who were referred to nearby health facilities in their communities were also followed up to ensure that they reached the facility. One of the respondents said, “We do follow up. We go to those health centers and find out whether this client reached. We also have referral systems and telephone numbers of different facilities, so we do make calls to find out whether they’ve reached us. Linkage facilitators are also sent to nearby health facilities to confirm if the clients reached. If we get the contact address of the person, we try to follow through with phone calls or even home visits.” A 27-year-old female, counselor.

### 3.8. Psychosocial Support.

Psychosocial support for seropositive people has the potential to address the psychological and social problems that HIV-infected people, their partners, families, and caregivers face. Most of the key informants stated that involving family members provided clients with psychosocial and emotional support. In addition, this is particularly relevant in circumstances where seropositive clients refuse initiation into ART care. Under such circumstances, the combined efforts of close associates and counselors were considered necessary to help clients get initiated into care. One of the key informants said that

“If somebody objects based on their fear, you get some close relatives and then you involve them. You sit down with them, you talk to them together with the client, and he/she will be supported and start taking the medicine.” 35-year-old male, counselor.

### 3.9. Community ART Initiation.

Carrying drugs and registers to outreaches ensured immediate and early initiation of care. This innovation is essential for improving the level of linkage following community diagnosis, optimal disease management, and the promotion of health. In addition, the registers help in capturing client information that would help in follow-up after ART initiation. The community ART initiation also helped reduce the burden of transport costs and lessen the loss of clients after diagnosis. One of the clinical officers said that “We usually carry drugs and our register to the community so that the client who accepts to be initiated on ART is initiated there and then.” A 27-year-old female, counselor.

Following community-level identification of HIV-positive individuals, three themes were identified as barriers to linkage to care.

### 3.10. Denial.

Denial is a very common reaction following an HIV diagnosis. This is likely to hamper early linkage to HIV care following diagnosis. The key informants reported that some clients, mostly the youths, were not accepting their results, saying their results were false and thus they were not easily linked to care. Some of the seropositive clients did not believe their results because they were tested at outreaches. They believed that only facility-based testing would give them their correct results.

“Some of these clients deny their outcomes, with the youth bearing the brunt of this denial.” A 48-year-old female, linkage facilitator.

“…. Some may complain that I am not confirmed, have been tested in the market, have been tested in the outreach, have been tested as an APN, so I am not yet certain about my status.” A 66-year-old female, linkage facilitator.

### 3.11. Fear of Stigma.

Participants frequently said that some clients are not easily linked to care because most of them fear social rejection. They thought they would be isolated or rejected by others and therefore did not want to be seen going to the health facility for treatment. They would, therefore, rather not take their medications than suffer humiliation from friends, relatives, and neighbors, among others. A linkage facilitator said, “…Some fear their neighbors’ seeing them taking drugs.” A 26-year-old female, linkage facilitator.

### 3.12. Fear of Disclosure.

Some clients were not willing to disclose their status. Those who were married feared they would be divorced. They therefore preferred not going to the facility or taking drugs and, as a result, were not easily linked to care.

“…. Some because of their partners, they do not want their partners to get to know they are on treatment, when you link them, they may disagree to go with the drug.” A 42-year-old male, linkage facilitator.

In addition, another key informant also said, “…. Because you could find someone who has a partner and he or she tests positive, and she fears that if I go back with these drugs, what will my partner tell me? So, at times, if they are told they are positive, they just walk away and refuse to be linked. (at is a big challenge we normally face.” A 22-year-old male, linkage facilitator.

## 4. Discussion

The success of the “test and treat” approach towards increasing HIV testing uptake and treatment of identified cases is heavily reliant on attaining high levels of linkage to care following HIV diagnosis. In our study, linkage to HIV care following CB-HCT was nearly universal (98%) within 6 months of diagnosis for a sample of 320 participants recruited through three participating health facilities in Lira district, Uganda. These findings show a notably higher level of linkage than other comparable studies done in similar settings. Our findings show a higher level of linkage to HIV care compared to the evidence from Tanzania, where linkage was only 68% [19]. This could have been due to the activities of the implementing partners like RHITES North Lango, which have been promoting the adoption of healthy behaviors and raising awareness at the individual, provider, and community levels, with the goal of reducing delays in seeking care and lowering barriers to service. This could have provided an incentive, which could have encouraged linkage in these rural communities. In addition, a 2018 meta-analysis of studies done in sub-Saharan Africa on linkage to care following CB-HCT showed that levels of linkage ranged from 10% to 79% [14]. In other settings, linkage to care at individual levels was influenced by fear of not being able to continue with livelihood if one’s status were to be known [23].

In our study, the high level of linkage observed was found to be associated with self-initiated testing as compared to provider-initiated testing. We further found out that participants who did not perceive the distance to the health facility as a barrier were less likely to self-initiate the HIV testing and link to care. This finding is consistent with results from other settings that found distance to the health facility as a contextual factor in linkage to care [19].

On the other hand, our study found that the client’s perception that waiting time at health facilities is not a barrier increases the likelihood of linkage to HIV care compared to their counterparts. The perception that waiting time at health facilities is not a barrier may encourage clients to travel to the facilities and patiently wait to be served. This finding of our study from the northern Ugandan setting is consistent with evidence from rural Tanzania and Central Uganda, where long waiting times were reported as a barrier to linkage to HIV care [19, 24].

The higher levels of linkage may have been attributed to the additional strategies that were employed by healthcare providers to facilitate immediate linkage/initiation of ART through community ART initiation. During the interviews, some of the healthcare providers disclosed that they carried HIV medications and ART registers to HIV community outreach activities, such that those who tested positive and agreed to initiate ART immediately received their medications. Some randomized trials have shown that same-day ART initiation during a patient’s first clinic visit can reduce the likelihood of loss to care in the period before ART initiation [25]. Other studies have shown that enrolling newly diagnosed persons on-site (home, work, or any convenient place in the community) cut transport costs and also brought access to treatment to those who had time constraints on traveling to the referral facility due to work or other commitments [26].

In addition to community ART initiation, it is also likely that the HIV-prevention education that is given during community outreach activities has had a positive impact on communities. The National HIV Prevention Strategy in Uganda through the Ministry of Health and several implementation partners has in recent years promoted service provision alongside education, addressing sociocultural and behavioral issues that facilitate the spread of HIV, as well as prevention education. Health education plays a major role in ensuring that patients are aware of their health status, available medical services, and options for treatment in order to make informed decisions. Key informants (6/10) who were interviewed in this study also agreed that creating awareness through giving health talks enabled clients to make better health choices and was a likely factor in the increasing rates at which clients linked to care.

Concerning the factors associated with linkage to HIV care that were explored in this study, the majority of respondents reported transport costs as a major barrier to linkage. Those who perceived distance to healthcare facilities as a barrier were less likely to be linked to care. Previous studies have also reported transport costs and travel distance to care facilities as barriers to HIV care [27, 28]. While HIV services have been available in the country at no cost, these findings suggest that efforts to improve linkage to these services should prioritize bringing services closer to clients in the communities to mitigate this potential barrier.

In addition to health education and community/on-site ART initiation, other themes that emerged from the healthcare providers’ perspectives included HIV care counseling, follow-up, and family support. The perceived experience of healthcare providers was that newly diagnosed people were more likely to link to HIV care when they received the necessary counseling, and their linkage was facilitated, for example, through phone calls or follow-up at the referral facilities. These findings are consistent with results from a randomized control trial that achieved high linkage rates when follow-up visits were made by a lay counselor to facilitate linkage [9]. Healthcare providers also emphasized the role of psychosocial support for people living with HIV as it has the potential to address the psychological and social problems of HIV-infected individuals, their partners, families, and caregivers. Most of the key informants indicated that involving family members provided clients with psychosocial and emotional support. This is particularly relevant in circumstances where HIV-positive people refuse initiation into ART care. Under such circumstances, combined efforts from close associates and counselors were considered necessary to help clients get initiated into care.

Healthcare providers’ perceived barriers to linkage to HIV care included social distresses such as denial, fear of stigma, and fear of disclosing one’s HIV status. As observed in other studies [29, 30], HIV-related stigma continues to affect the care-seeking behavior of people living with HIV. Fear of the potential consequences of HIV-related stigma, for example, discrimination, abandonment, or even partner violence, prevents some clients from seeking care. These perspectives emphasize the central role of social support and the need to fight HIV-related stigma at individual, household, and community levels. According to Bogart and others [29], the providers believed that it was the responsibility of the clients to overcome stigma within themselves.

### 4.1. Study Limitations.

Our study was conducted in public health facilities, and the findings may not be generalizable to the private health facilities. We think that some information regarding linkage to care might not have been captured. Future studies should study the linkage between public and private health facilities in order to better inferences regarding linkage following community-level identification. We considered clients who tested positive for HIV within 6 months prior to the suspension of community-based testing due to the effects of COVID-19. The clients may have had more time to initiate care, and this is likely influenced by the higher linkage observed.

## 5. Conclusion and Recommendations

We found a high level of linkage to HIV care following community-based HCT among the HIV-positive cases in a remote rural setting of Northern Uganda. The independent predictors of successful linkage were self-initiated testing, perception that distance to the health facilities and waiting time are not a barrier. Our study resonates with previous findings and builds on existing literature to highlight the comparative success of community-based HIV testing as a scalable alternative to achieving universal testing and treatment in resource-poor settings. The benefits of community-based HCT could be augmented by integrating testing with on-site community ART initiation or other HIV service delivery models that bridge the physical distance between patients and HIV care services. Efforts to improve self-initiation of HIV testing, positive perceptions of distance, and waiting time at health facilities through awareness raising, health education, and counselling would increase linkage to HIV care.

## Figures and Tables

**Table 1:** Sociodemographic characteristics of the respondents.

Variables	Frequency (*n* = 329)	Percentage (%)
Sex of respondents		
Females	233	70.8
Males	96	29.2
Age of respondents (years)		
18–35	100	30.4
36–53	169	51.4
54–85	60	18.2
Marital status		
Single	15	4.6
Married	193	58.7
Divorced	45	13.7
Widowed	76	23.0
Highest education level attained		
No education	87	26.4
Primary	204	62.0
Secondary plus	38	11.6
Occupation of the respondents		
Farmer	282	85.7
Business and civil servants	47	14.3

**Table 2:** Association of sociodemographic characteristics and linkage to care following community-level identification.

Variable	Linkage to care		COR	95% CI	*p* value
	Yes 320 (%)	No 9 (%)			
Sex					
Male	92(28.8)	4(44.4)	1.00		
Female	228(71.2)	5(55.6)	1.98	0.52–7.55	0.32
Age categories					
18–35	99(31.0)	1(11.1)	1.00		
36–53	162(50.5)	7(77.8)	0.23	0.03–1.93	0.18
54–85	59(18.5)	1(11.1)	0.60	0.04–9.71	0.72
Marital status					
Single	131(40.9)	5(55.6)	1.00		
Married	189(59.1)	4(44.4)	1.80	0.48–6.84	0.39
Level of education					
None	86(26.9)	1(11.1)	1.00		
Primary	199(62.2)	5(55.6)	0.46	0.05–4.02	0.49
Secondary and above	35(10.9)	3(33.3)	0.14	0.01–1.35	0.09
Occupation					
Farmer	275(85.9)	7(77.8)	1.00		
Business and civil servants	45 (14.1)	2(22.2)	1.18	0.14–9.64	0.88

**Table 3:** Personal factors associated with linkage to care following community-level identification.

Variable	Linkage to care		Crude odds ratio	95% CI	*p* value
	Yes *n* (%) No *n* (%)				
Receiving ART outside Lira district					
No	294 (91.9)	8 (88.9)	1.0		
Yes	26 (8.1)	2 (11.1)	0.7	0.085–5.878	0.75
Respondent currently having a main sexual partner					
No	194 (60.6)	4 (44.4)	1.0		
Yes	126(39.4)	5 (55.6)	0.5	0.137–1.972	0.34
Used a condom during last sexual activity					
No	260 (81.8)	7 (77.8)	1.0		
Yes	58 (18.2)	2 (22.2)	0.8	0.158–3.856	0.76
Ever received HIV information from outreach					
No	170 (53.1)	6 (66.7)	1.0		
Yes	150 (46.9)	3 (33.3)	1.8	0.434–7.179	0.43
Clients received posttest counseling					
No	11 (3.4%)	0(0)	1.0		
Yes	309 (96.6%)	9(100)	34	17.696–66.611	≤0.001***
Clients perceived distance to the facility as a barrier					
Barrier	205 (64.1)	4 (44.4)	1.0		
Not a barrier	115 (35.9)	5 (55.6)	0.4	0.118–1.704	0.24
Clients perceived cost of transport as a barrier to receiving care					
Barrier	215 (67.2)	6 (66.7)	1.0		
Not a barrier	105 (32.8)	3 (33.3)	1.0	0.240–3.982	0.97
Clients perceived health workers as unfair					
Barrier	26 (8.1)	2 (22.2)	1.0		
Not a barrier	294 (91.9)	7 (77.8)	3.2	0.638–16.355	0.16
Clients considered timeliness of HIV care services as a barrier					
Barrier	186 (88.9)	8(11.1)	1.0		
Not a barrier	134 (58.1)	1(41.9)	5.8	0.712–46.629	0.101

*** refers to or means the *p*-value <0.001.

**Table 4:** Predictors of linkage to HIV following community-level diagnosis.

Linkage	Adjusted odds ratio	95% confidence interval	*p* value
Education level of the respondents			
None	1.00		
Primary	0.37	0.040–3.488	0.388
Secondary	0.03	0.002–0.451	0.011
Occupation of the respondents			
Peasant farmer	1.00		
Business and civil service	2.09	0.189–23.063	0.549
Clients self-initiated the testing process			
No	1.00		
Yes	9.03	1.271–64.218	0.028
Client received pretest counseling			
No	1.00		
Yes	1.37	0.296–6.305	0.69
Client has a committed sexual partner			
No main partner	1.00		
Has a main sexual partner	0.28	0.060–1.263	0.097
Client received health education in the community			
Did not receive	1.00		
Received health education	2.70	0.566–12.867	0.213
Client perceived waiting time at the health facilities as a barrier			
A barrier	1.00		
Not a barrier	10.39	1.119–96.563	0.04

## Data Availability

Data can be made available upon reasonable request.
